# Supplementary material for: Reversible non-volatile electronic switching in a near-room-temperature van der Waals ferromagnet
Source: Nat Commun. 2024 Mar 28;15:2739. doi: 10.1038/s41467-024-46862-z (PMC10978849; doi:10.1038/s41467-024-46862-z)
Supplement: Supplementary file 1 — Supplementary Information [file 41467_2024_46862_MOESM1_ESM.pdf]

Supplementary Information

(Reversible non-volatile electronic switching in a near-room-temperature van der Waals ferromagnet)

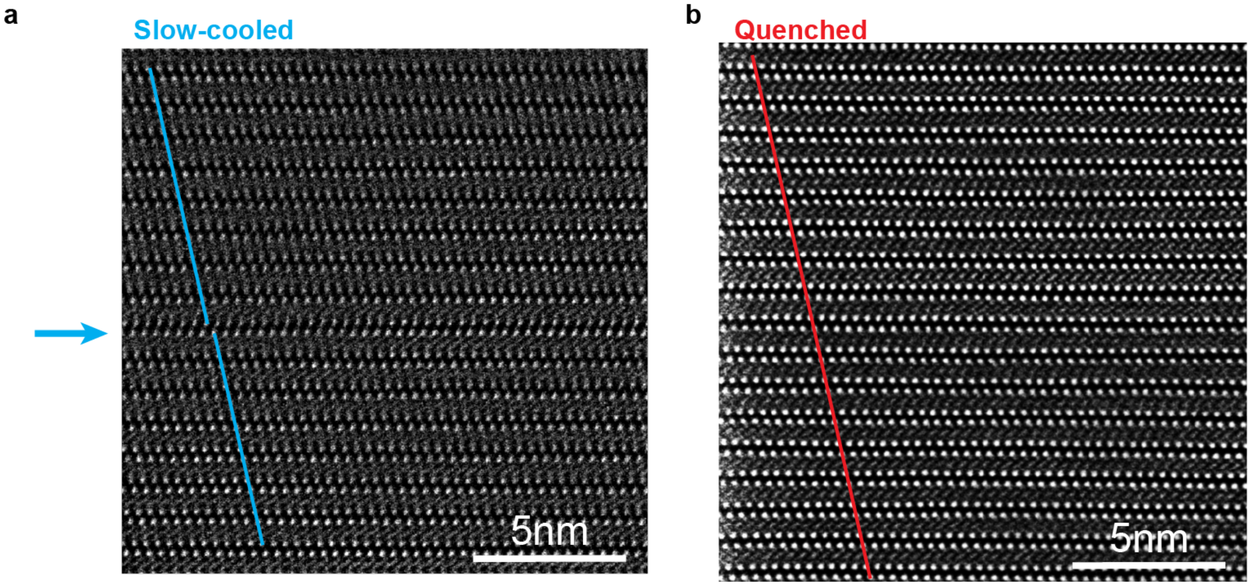

FIG. **Supplementary Figure 1**. **TEM image of $Fe_{5-\delta}GeTe_2$ cross section.** **a** Annular Dark-Field Scanning Transmission Electron Microscopy (ADF-STEM) images of a slow-cooled $Fe_{5-\delta}GeTe_2$ sample along the [100] direction, showing ABC stacking of the vdW slabs. The blue arrow points to a stacking fault between vdW slabs. **b** The same measurement on a quenched $Fe_{5-\delta}GeTe_2$ sample, showing the ABC stacking of the vdW slabs.

SUPPLEMENTARY NOTE 1: TRANSMISSION ELECTRON MICROSCOPY CHARACTERIZATION

The TEM image (Supplementary Figure 1) indicates the layered structure of $Fe_{5-\delta}GeTe_2$. The most bright points are from Te atoms, a ABC stacking order were revealed by comparing each layers Te atoms' position, seeing the guide line for slow-cooled and quenched samples. A stacking fault was found between the vdW sublayers in slow-cooled sample pointed by the blue arrow. This result is consistent with previous report that the slow-cooled sample show more stacking faults [1, 2].

SUPPLEMENTARY NOTE 2: SHG MEASUREMENTS AND SYMMETRY ANALYSIS

We derive the equation of SHG intensity vs. incident polarization in slow-cooled and re-quenched $Fe_{5-\delta}GeTe_2$ crystals, by considering the electric quadrupole (EQ) SHG term $I_i^{\mathrm{EQ}}(2\omega) \propto \left|\Sigma_{\mathrm{jkl}}\chi_{\mathrm{ijkl}}^{\mathrm{EQ}} k_j E_k(\omega) E_l(\omega)\right|^2$ and the inversion symmetry breaking induced electric dipole (ED) term $I_i^{\mathrm{ED}}(2\omega) \propto \left|\Sigma_{\mathrm{jk}}\chi_{\mathrm{ijk}}^{\mathrm{ED}} E_j(\omega) E_k(\omega)\right|^2$, respectively. The two tensors $\chi_{\mathrm{ijkl}}^{\mathrm{EQ}}$ and

$\chi^{\text{ED}}_{\text{ijk}}$ must agree with the symmetry presented in these crystals.

Slow-cooled crystals

The slow-cooled crystal has the point group $\overline{3}m$ and space group $R\overline{3}$m (166), which preserves the inversion symmetry and has $\chi^{\text{ED}}_{\text{ijk}} = 0$. In that, the ED SHG term vanishes, while a smaller EQ SHG term may be present. Under normal incidence, one can simplify the EQ SHG term $\chi^{\text{EQ}}_{\text{ijkl}} k_j E_k(\omega) E_l(\omega)$ by restricting $k, l \; = \; x, y$ and fixing $j = z$. According to the symmetry of the EQ SHG tensor $\chi^{\text{EQ}}_{\text{ijkl}}$, one can write the SHG intensity as:

$$I_{\text{parallel}}(2\omega) \propto \left(\chi^{\text{EQ}}_{\text{xzxx}}\right)^2 I(\omega)^2 \cos^2 3\alpha \tag{1}$$

$$I_{\text{crossed}}(2\omega) \propto \left(\chi^{\text{EQ}}_{\text{xzxx}}\right)^2 I(\omega)^2 \sin^2 3\alpha \tag{2}$$

where α is the angle between the input polarization and the crystalline x axis and $I(\omega)$ is the incident power. From the above equations, one can see both parallel and crossed configuration has six lobes with equal intensity in their polar pattern, matching well with our observation (Fig. 1i).

Quenched crystals

In the re-quenched crystals, the formation of the $\sqrt{3} \times \sqrt{3}$ superstructure breaks inversion symmetry, which allows a non-zero ED contribution to SHG intensity. We used the DUU structure as an example to derive the polarization dependent SHG intensity. A mirror plane is found in the structure. The mirror plane is parallel to the c axis and parallel to the bisector of the angle formed by a and b axis. This matches the point group $3m$. It holds point group $3m$, which will give the corresponding SHG polar pattern under parallel and crossed configuration:

$$I_{\text{parallel}}(2\omega) \propto \left(\chi^{\text{ED}}_{\text{xxy}}\right)^2 I(\omega)^2 \sin^2 3\alpha \tag{3}$$

$$I_{\text{crossed}}(2\omega) \propto \left(\chi^{\text{ED}}_{\text{xxy}}\right)^2 I(\omega)^2 \cos^2 3\alpha \tag{4}$$

where $I(\omega)$ is the input power and α is the angle between crystalline x axis and input polarization. Thus, both parallel and crossed setup will give six lobes with the same amplitude in their polar pattern, agreeing with our experimental results (Fig. 1j).

SUPPLEMENTARY NOTE 3: SINGLE-CRYSTAL DIFFRACTION

We compare the diffraction data at the L=0 scattering plane for the two types of samples. Since cutting a single crystal for vdW materials creates edges, we instead take two slow-cooled samples from the same batch and carefully re-quenched one prior to XRD measurements. Supplementary Figure 3a shows the slow-cooled single crystal XRD pattern and Supplementary Figure 3b shows the data from the re-quenched crystal. Comparing the two sets of data, the slow-cooled sample shows stronger disorder from the presence of many ring-like features. In addition, the presence of the $\sqrt{3} \times \sqrt{3}$ superstructure peaks indicative of the Fe(1) site ordering is present in both crystals. However, as we will demonstrate with STM, the $\sqrt{3} \times \sqrt{3}$ order appears in slow-cooled crystals as small puddles. To compare the superstructure population difference between the two types of samples, we pick a region in the scattering plane free from ring-like disorder (see boxed region in Supplementary Figure 3). To capture the entire peak with finite width, we integrate along the shorter width of the boxes and compare the integrated line profile for the two types of samples as shown in Supplementary Figure 3c, where we have normalized the curves by the Bragg peak intensity. The area of the superstructure peak is larger in the quenched sample indicating that the population of $\sqrt{3} \times \sqrt{3}$ ordered region is larger in the quenched crystals, which is consistent with previous reports [1, 2].

SUPPLEMENTARY NOTE 4: SCANNING TUNNELING MICROSCOPY MEASUREMENTS AND ANALYSIS

Supplementary Figure 2 shows the STM topography images of four types of regions we have found, along with the Fourier transforms of each respective topographies as the insets. Supplementary Figure 2b has pixel density of 6.61 pixels/nm. Supplementary Figure 2c-e have pixel density of 24.8 pixels/nm. The peaks in the Fourier transforms correspond to the lattice periodicity (white), a $\sqrt{3} \times \sqrt{3}$ superstructure periodicity (blue), and the second

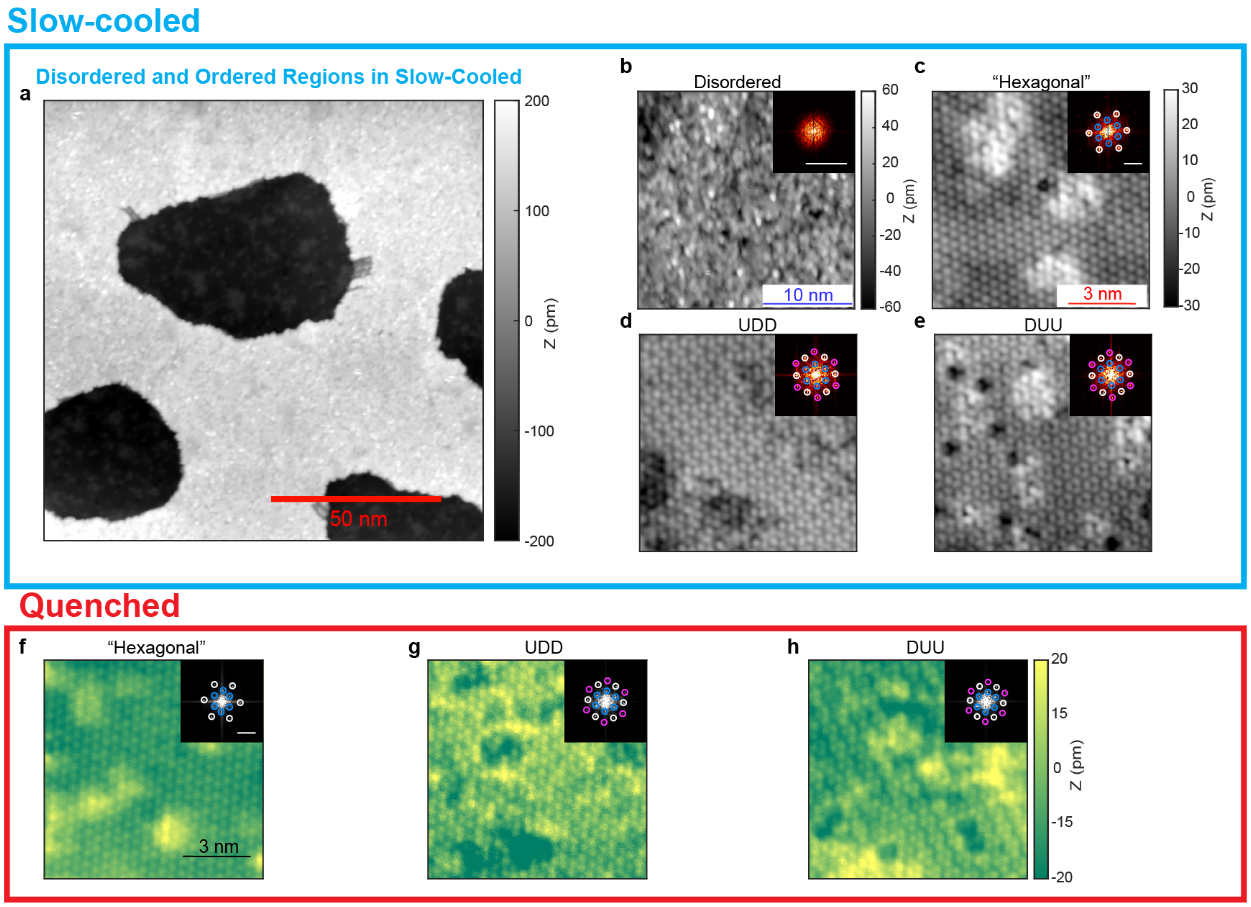

FIG. **Supplementary Figure 2**. **STM topographies of $Fe_{5-\delta}GeTe_2$. a** STM topography on a Te termination in a slow-cooled $Fe_{5-\delta}GeTe_2$ sample. The lighter region is a disordered surface, surrounding the darker regions that contain the three ordered surfaces (hexagonal, UDD, DUU). **b-e**, Different STM topographies on a Te termination in a slow-cooled $Fe_{5-\delta}GeTe_2$ taken on a disordered surface as shown in **a** and three ordered surfaces: an apparently unmodulated hexagonal lattice as shown in **c**, a lattice corresponding to an up-down-down (UDD) ordering of subsurface Fe(1) atoms as shown in **d**, and a lattice corresponding to a down-up-up (DUU) ordering of subsurface Fe(1) atoms as shown in **e**. **f-h** STM topographies on a Te termination in a quenched $Fe_{5-\delta}GeTe_2$ sample, taken on three ordered surfaces: **f** an apparently unmodulated hexagonal lattice, **g** a lattice corresponding to an UDD ordering of subsurface Fe(1) atoms, and **h** a lattice corresponding to a DUU ordering of subsurface Fe atoms. The hexagonal regions in both samples likely correspond to the regions with Fe vacancies, with a crystal structure of Fe_4GeTe_2 with no Fe(1).

order of the $\sqrt{3} \times \sqrt{3}$ superstructure periodicity (pink). The inset scale bars are 17.3 nm^{-1}. Topographies in Supplementary Figure 2c-e are of the same size.

In the slow-cooled sample, we find the cleaved surface to be dominated by a disordered region within the field of view as plotted in Supplementary Figure 2a. The larger field of view in Supplementary Figure 2a was measured with set-point current of 50 pA and bias of 100 mV. The disordered topography in Supplementary Figure 2b was measure with a

set-point current of 200 pA and bias of 200 mV. In addition, we also found the presence of the three types of ordered surfaces formed as small puddles of typically 50 nm within the disordered region Supplementary Figure 2c-e. Each ordered topography was measured with set-point current of 1 nA and bias of 25 mV. The hexagonal surface Supplementary Figure 2c is likely a Fe(1) vacancy region (Fe_4GeTe_2) corresponding to the Fe deficient regions. In addition, we also observe regions that correspond to the previously identified UDD and DUU regions Supplementary Figure 2d-e whose Fourier transforms exhibit a $\sqrt{3} \times \sqrt{3}$ periodicity from a up-down-down or down-up-up ordering of the subsurface Fe(1) atoms [3]. For the quenched crystal, within the field of view, we only observed the presence of the three ordered surfaces (Hexagonal, UDD and DUU).

To compare the difference between the quenched and slow-cooled crystal, we annealed and quenched the same batch of the slow-cooled sample and check the cleaved surface by STM. As shown in the Supplementary Figure 2f-h, we did not find any disordered region in the quenched crystal and only three ordered region are found in the field of view. Each topography in Supplementary Figure 2f-h was measured with set-point current of 1.2 nA and bias of 25 mV. Such result suggesting the UDD and DUU region is more dominating in the quenched crystals.

SUPPLEMENTARY NOTE 5: PHOTON ENERGY DEPENDENCE AND ADDITIONAL ARPES DATA

We carried out photon-energy dependence measurements to probe the electronic structure along the out-of-plane direction for the two types of crystals. The quenched crystal was directly quenched from growth, while the slow-cooled crystal was slow-cooled from growth. We measured the $\bar{K}$–$\bar{\Gamma}$–$\bar{K}$ direction of both types of crystals as a function of photon energy. For the slow-cooled crystal (Supplementary Figure 6a), the measurements were taken with LV polarized photons with the photon energy ranging from 60 to 164 eV. For the quenched crystal (Supplementary Figure 6b), the photon energy range was from 30 to 180 eV. Due to the 2D nature of the vdW material, we do not observe strong variation of the dispersions along the k_z direction, preventing us from accurately determining the inner potential. The k_z maps shown in Supplementary Figure 6a-b were produced with an inner potential of 17 eV to demonstrate that we have covered multiple BZs along k_z.

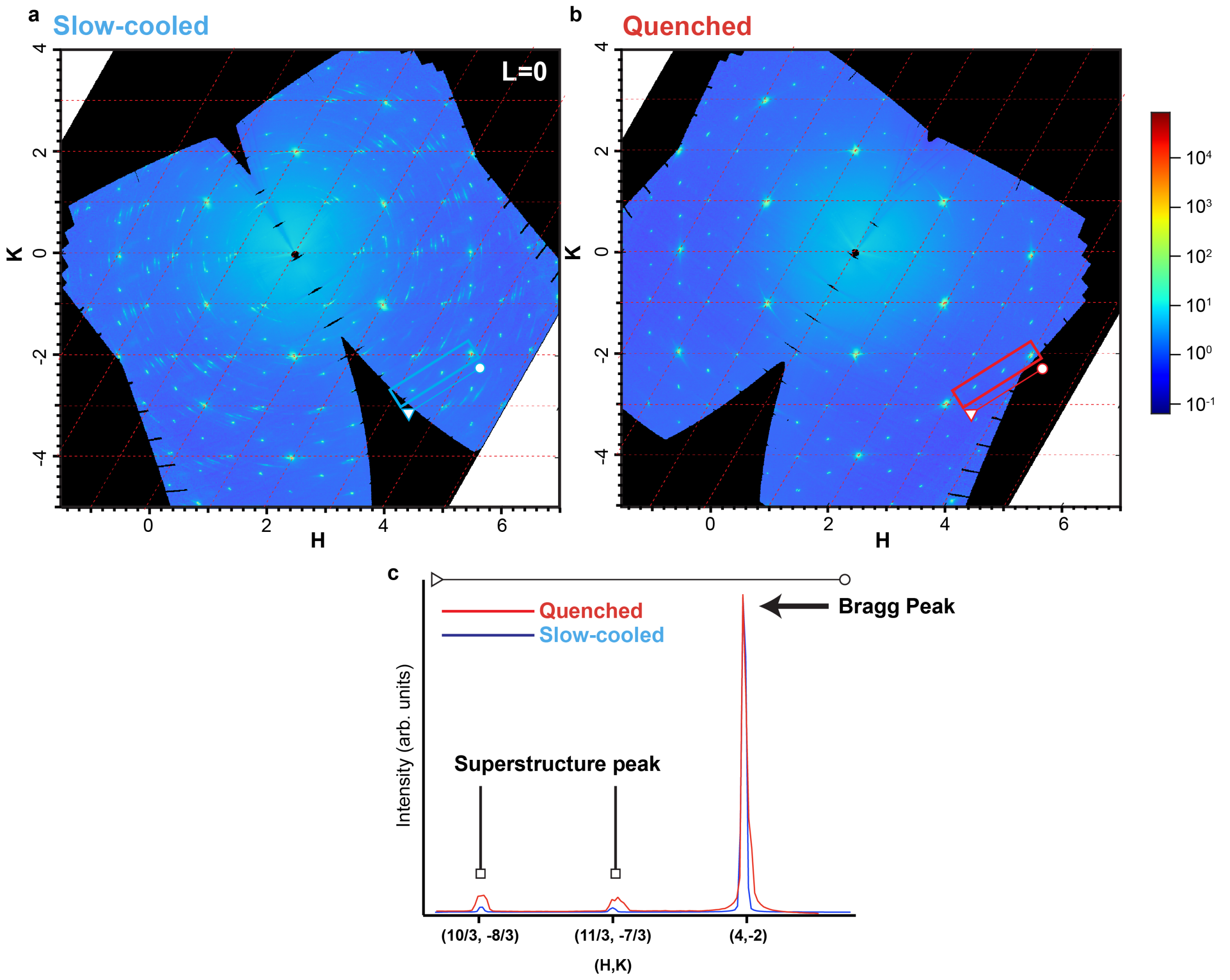

FIG. **Supplementary Figure 3**. **Single crystal x-ray diffraction. a-b**, Single crystal x-ray diffraction data from the (HK0) scattering planes in a **a** furnace slow-cooled sample and **b** a sample requenched from the same batch of samples as **a**. **c**, Line profile from the boxed region shown in **a-b** where the shorter width of the boxes were integrated. the black arrow points to the Bragg peak. The superstructure peaks are also indicated. The data were taken at 300K.

We summarize additional Fermi surface mapping and band dispersions along the $\bar{K}$–$\bar{\Gamma}$–$\bar{K}$–$\bar{M}$ direction measured with 114 eV and 132 eV photons in Supplementary Figure 6. The topological crossing near -0.2 eV at K are clearly shown in slow-cooled site-disordered crystals under LV polarization. For the quenched Fe(1) site-ordered samples, crossings at K point are no longer observed, consistent with the broken inversion symmetry. Instead, three flat bands are clearly observed under LH polarization across most of the BZ and hybridized with dispersive bands.

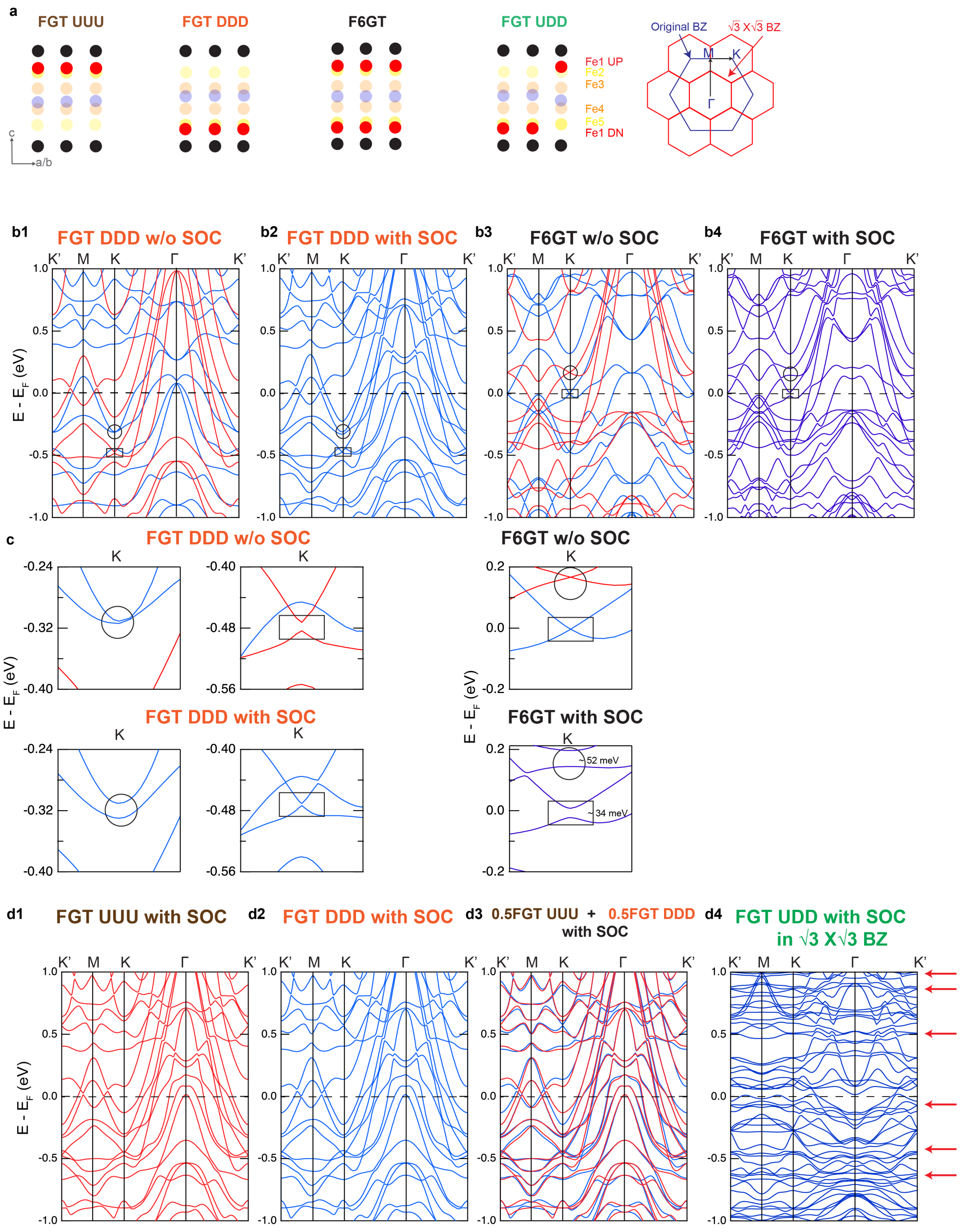

FIG. **Supplementary Figure 4**. **DFT calculations a** Definition of lattice structures used for DFT calculations and the BZ. **b1-b2** DFT calculations for Fe_5GeTe_2 with all up Fe(1) sites occupied, without SOC and with SOC. **b3-b4** DFT calculations for Fe_6GeTe_2 with all Fe(1) sites occupied, without and with SOC. In this case, the inversion symmetry is preserved. **c** Zoomed in view of bands near K for **b1-b4**. Only in the inversion-symmetry preserving Fe_6GeTe_2 do we see protected crossings that become gapped when SOC is included. **d1-d2** DFT calculations for UUU and DDD with SOC. **d3** To simulate the site-disordered case, the UUU and DDD calculated band structures are overlapped. **d4** DFT calculations of UDD Fe_5GeTe_2 plotted in the small folded BZ defined in **a**. Some potential flat bands are marked with red arrows.

SUPPLEMENTARY NOTE 6: MIXED PHASES

While for all the crystals that we measured with ARPES, either the topological nodal line or flat band dispersions were observed following a slow-cooling or quenching, respectively, we did observe one case resembling the mixing of the two phases (Mixed State). The data is presented in (Supplementary Figure 7a,d), which is compared to the topological nodal line phase (Supplementary Figure 7b,e) and the flat band phase (Supplementary Figure 7c,f). The mixed state shows both topological crossing at K point (blue dotted lines) as well as the flat bands (red dotted lines). Even the dispersive hole bands at the BZ center are an overlap of the two phases. As the ARPES beamspot is 50 μm^2, this mixed signal is most compatible with the understanding that the sample contains both regions of site-disordered occupation of Fe(1) and regions with $\sqrt{3} \times \sqrt{3}$ superstructure. This mixing is consistent with the understanding that these are the two most stable formations of the Fe(1) sites, which always exist in domains and the quenching or slow cooling processes modify the balance of the different domain populations, as also demonstrated by the coexistence of the two phases by XRD and STM measurements. We also note that the coexistence of the two domains does not seem to affect the stability of the topological crossings of the site-disordered region as the crossings can still be observed in the crystal with mixed populations.

SUPPLEMENTARY NOTE 7: FLAT BAND ESTIMATION

We can estimate the flatness of the flat dispersions in the site-ordered region. Supplementary Figure 14 shows the constant energy contours taken at the energy of the three flat dispersions discussed in the main text. Three cuts are also shown for each set, from which we have fitted the disperions of the flat bands. The constant energy contours show shaded regions for the portions of the Brillouin zone where each flat band disperses within a 50 meV window. The estimated percentage of these regions are 40%, 84%, and 96% of the BZ for the flat bands at E_F, -0.22 eV, and -0.56 eV, respectively.

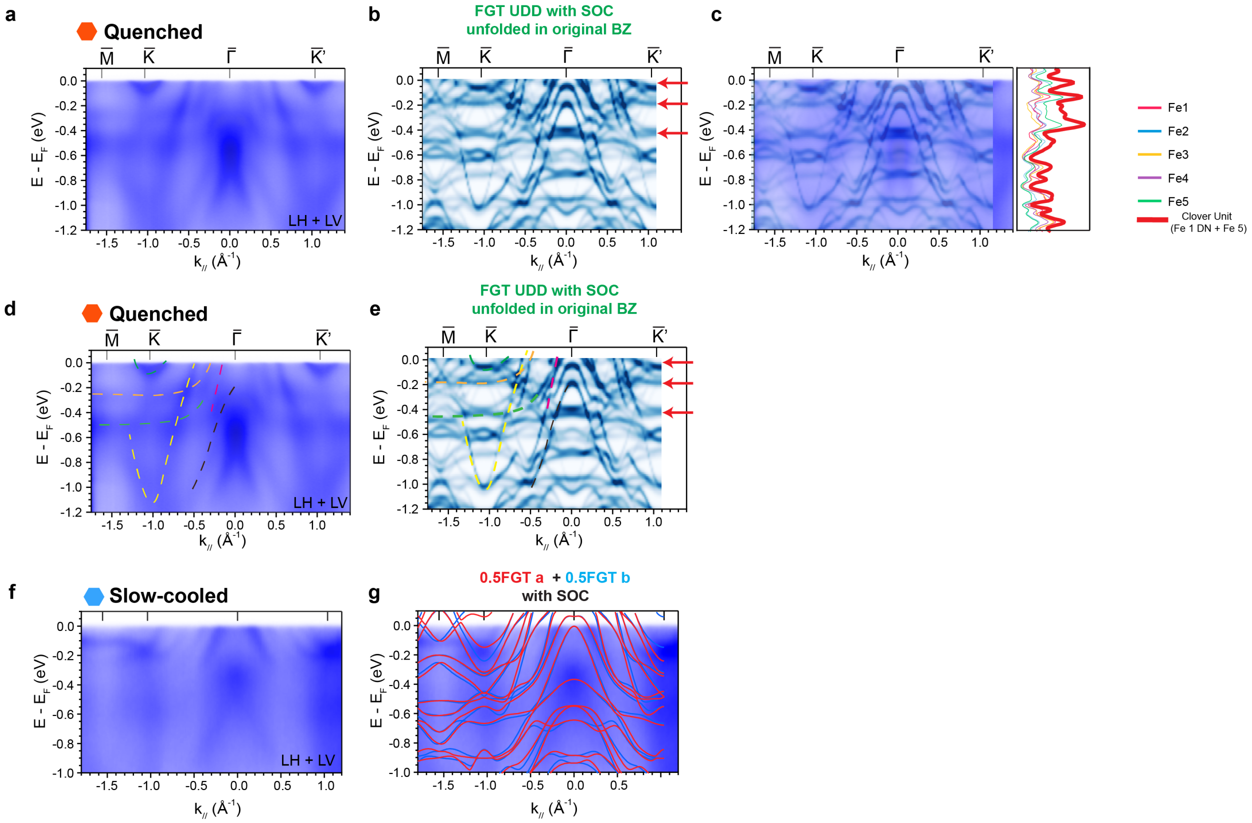

FIG. **Supplementary Figure 5**. **DFT calculations and ARPES data comparison.** **a**, ARPES measured dispersions of a quenched crystal. To visualize more bands, the spectra is from the sum of data taken under LH and LV polarizations. **b** DFT calculated band structure for the UDD FGT which is unfolded into the original BZ. **c**, Overlap of the ARPES data and DFT calculations and the density of state projection for different Fe sites. The flat band positions are marked with red arrows. **d-e** The same as **a-b** but with additional guides to the eye to show the similarity between the DFT and the data. We note that a chemical potential up-shift of 1eV is applied to all the UDD calculations shown here. **f-g** ARPES data of the slow-cooled crystal overlapped with 0.5 FGT UUU (red) + 0.5 FGT DDD (blue) calculations.

SUPPLEMENTARY NOTE 8: DENSITY FUNCTIONAL THEORY CALCULATIONS

Below we discuss two aspects of our DFT calculations. First we demonstrate the importance of the inversion symmetry to the topological band crossing at K. Second, we demonstrate the importance of the clover unit to the emergence of flat bands in the site-ordered phase. In order to do this, we have carried out DFT calculations for four types of crystal structures, as shown in Supplementary Figure 4. UUU represents the structure with all the up-Fe(1) sites occupied. DDD represents the structure with all the down-Fe(1) sites occupied, and is the

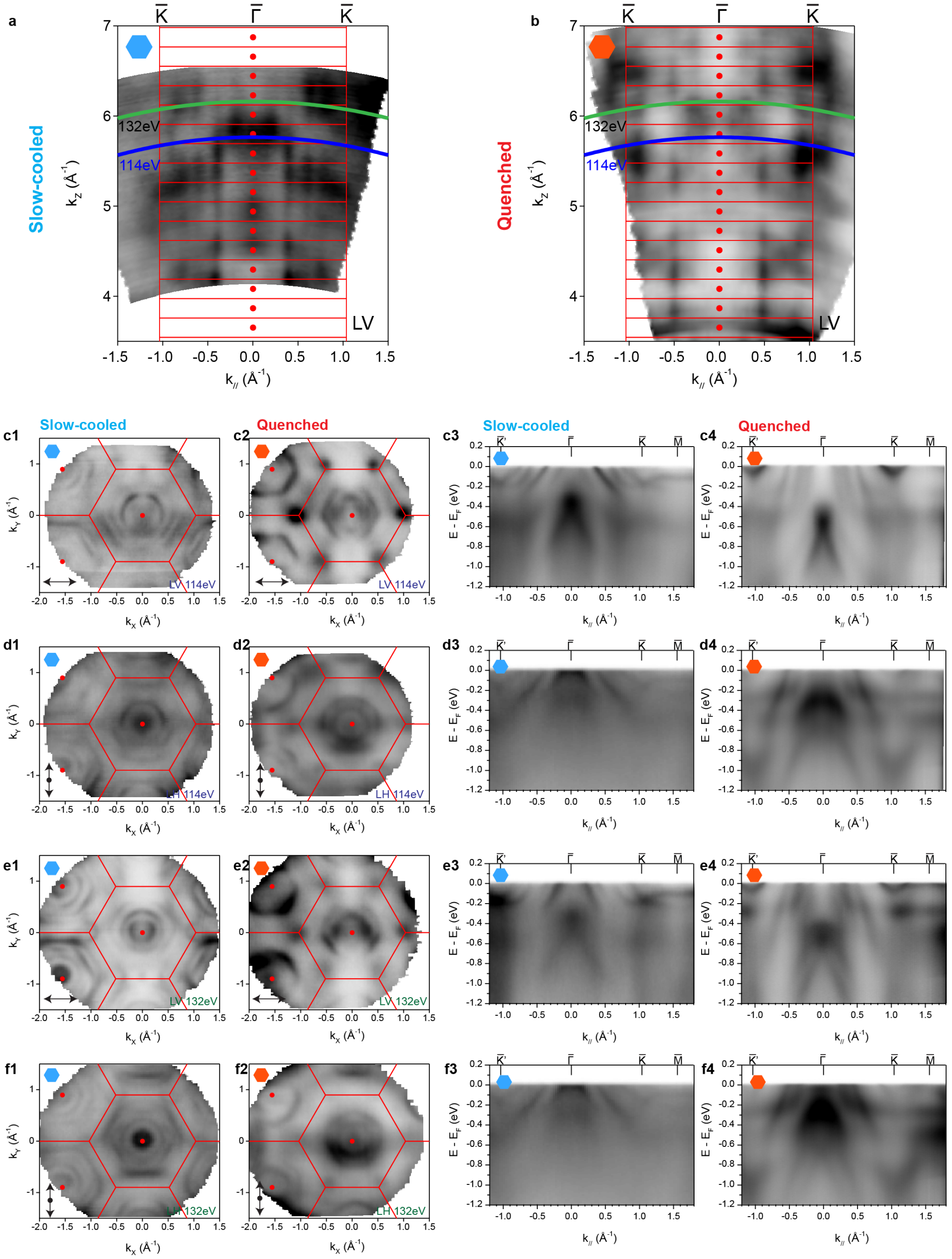

FIG. **Supplementary Figure 6**. **Additional ARPES data.** **a,b**, Photon energy dependent electronic structure of Fe(1) site-disordered state (blue hexagon) and Fe(1) site-ordered (red hexagon) state taken with LV polarization. **c1-c4**, Fermi surface mapping and corresponding high symmetry cut along K'–Γ–K–M direction taken with 114 eV LV light. **d1-d4**, Fermi surface mapping and corresponding high symmetry cut along K'–Γ–K–M direction taken with 114 eV LH light. **e1-e4**, Fermi surface mapping and corresponding high symmetry cut along K'–Γ–K–M direction taken with 132 eV LV light. **f1-f4**, Fermi surface mapping and corresponding high symmetry cut along K'–Γ–K–M direction taken with 132 eV LH light.

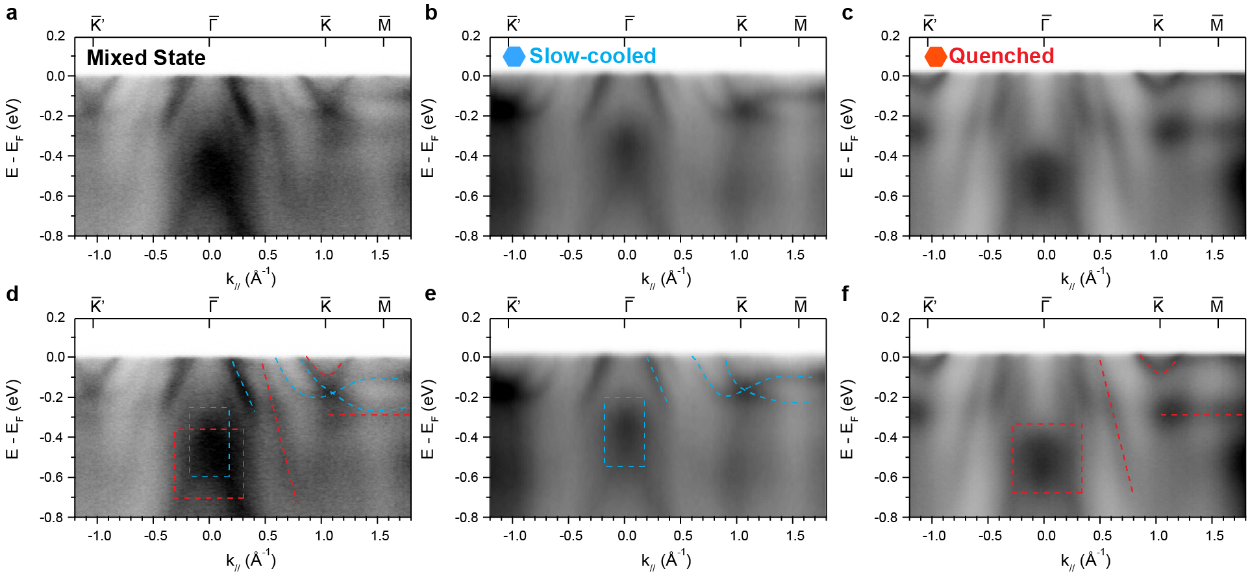

FIG. **Supplementary Figure 7**. **Mixed state and its comparison between Fe(1) site-disordered state and Fe(1) ordered state. a-c**, Mixed state, Fe(1) site-disordered state and Fe(1) ordered-site state electronic structure without any marker. **d-f**, the corresponding electronic structure with dashed line highlighting the key features in Fe(1) site-disordered state (blue) and site-ordered state (red). The dashed line marks the similarity between each state. All the data taken with the same geometry, temperature (15K) and photon energy (132 eV) and polarization (LV).

inverted structure of UUU. Both UUU and DDD have a stoichiometry of Fe_5GeTe_2. F6GT is the structure with all up and down Fe(1) sites occupied, and hence has a stoichiometry of Fe_6GeTe_2. Finally, UDD is the structure with the UDD order, and hence represents the site-ordered phase.

We first focus on the band crossings at K. As discussed and demonstrated in the main text, the inversion symmetry in the site-disordered phase is protected globally. The site-disordered Fe_5GeTe_2 has a random distribution of the Fe(1) sites with 50% chance to occupy the site above or below the Ge site. The UUU or DDD structure has the correct average stoichiometry. However, both structures have broken inversion symmetry. We can directly compare the band structure of these structure with that of the F6GT, which preserves inversion symmetry. This comparison is provided in Supplementary Figure 4b. As there appear to be near-crossings at K, we zoom-in to these regions in Supplementary Figure 4c and test these by comparing the calculations with and without SOC. We note that topological crossings protected by the three symmetries would become gapped by the inclusion of SOC. As can be seen, only the crossings in F5GT are real gapless crossings with SOC is not included, and develop gaps when SOC is included. The near-crossings for the DDD structure are not actual gapless

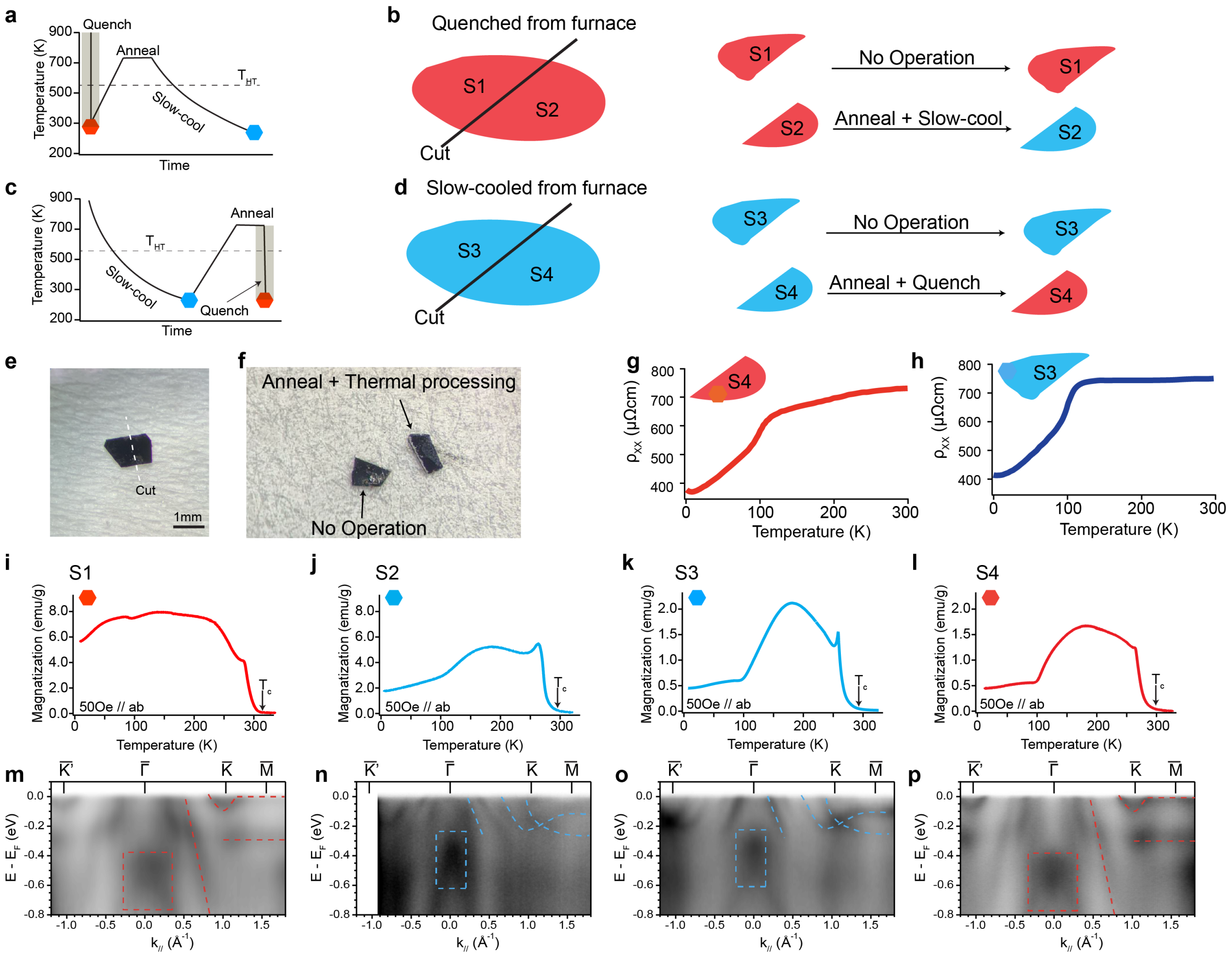

FIG. **Supplementary Figure 8**. **Demonstration of the switching of the electronic structure of $Fe_{5-\delta}GeTe_2$.** **a** Schematic view of thermal history and corresponding electronic state. The red hexagon means the Fe(1) ordered-site state while the blue hexagon means the Fe(1) site-disordered state. **b** Sequence of switching electronic state for a grown-quenched sample. The grown-quenched sample was cut into two pieces. The S2 was put into furnace for annealing and quenching. **c-d** Similar schematic and the experimental sequence for a grown-slow-cooled sample. **e-f** Real image of the experimental sequence in **d**. **g-h** The corresponding resistivity for the crystal that from the schematic in **d**. **i-l** The corresponding magnetization curves for S1-S4 as illustrated in **b,d**. **m-p** The summarized electronic structure of S1-S4. The red and blue dashed lines highlight the key features to identify the different electronic states. All data were taken at 15 K with 132 eV LV photons.

crossings even when SOC is not included. This comparison demonstrates that the inversion symmetry is important for the crossings in the site-disordered region. Moreover, we note that these crossings should be gapped when SOC is included. We cannot resolve the SOC gap clearly in our measured dispersions. This is likely due to a combination of energy resolution and moderate correlation effects associated with the Fe 3d states. Nonetheless, we have tried fitting for an upper bound of the gap size. As shown in Supplementary Figure 13, we have

fitted the energy distribution curves for a gap. The fit gives a peak separation of 14 meV, which is smaller than our energy resolution of 26 meV for this dataset. Hence the upper bound of the SOC gap is 26 meV. We also note that the SOC induced gap from the F6GT calculation is estimate to be 52 meV and 34 meV, which is on par with our estimation. Finally, we also compare the calculated band structure with our measured dispersions (Supplementary Figure 5g). This is done by overlapping both the UUU and DDD band structure on top of the measured dispersions to simulate an average of 50% occupancy of all the Fe(1) sites. We see that the overall comparison is reasonable except the lack of crossings at K in the calculation, due to the broken inversion symmetry of the UUU and DDD structures.

Next, we focus on the emergence of flat bands for the site-ordered phase. In Supplementary Figure 4d3-d4 we compare the band structure for the UUU+DDD structure (representing the site-disordered phase) and that for the UDD structure. Notably, with the same stoichiometry, flat bands appear in the site-ordered calculation. As the only distinction in the crystal structure is the Fe(1) site ordering, we ascribe these flat bands to the site-ordering that form the clove units. To compare this directly to the measured dispersions, we unfolded this band structure back to the original unfolded zone. We identify a number of flat bands in the unfolded spectral. However, to match with the experimentally observed dispersions, we have to shift the chemical potential of the calculation up by 1.0 eV. In Supplementary Figure 5d we identify the dispersions that give reasonable resemblance between the measured dispersions and the unfolded calculation, including hole-like dispersions around Γ, the electron band around the K point, and the flat bands. In Supplementary Figure 5c we also show the density of states projected unto the different Fe sites. In particular, we note that the flat bands have a large contribution by the Fe sites that constitute the clover unit.

SUPPLEMENTARY NOTE 9: SYMMETRY ANALYSIS AND EFFECTIVE MODEL FOR THE SITE-DISORDERED OCCUPATION

In this section, we focus on the case when the occupancy of the Fe(1) split-site is completely random. In this case, the lattice structure belongs to the space group $R\bar{3}m$ (SG166). The unit cell consists of three layers connected by the vdW interactions. The weak dispersion for all the bands along the k_z direction indicates that the interlayer vdW interaction is much smaller than the intra-layer interactions, as typical for vdW materials. As such, the analysis

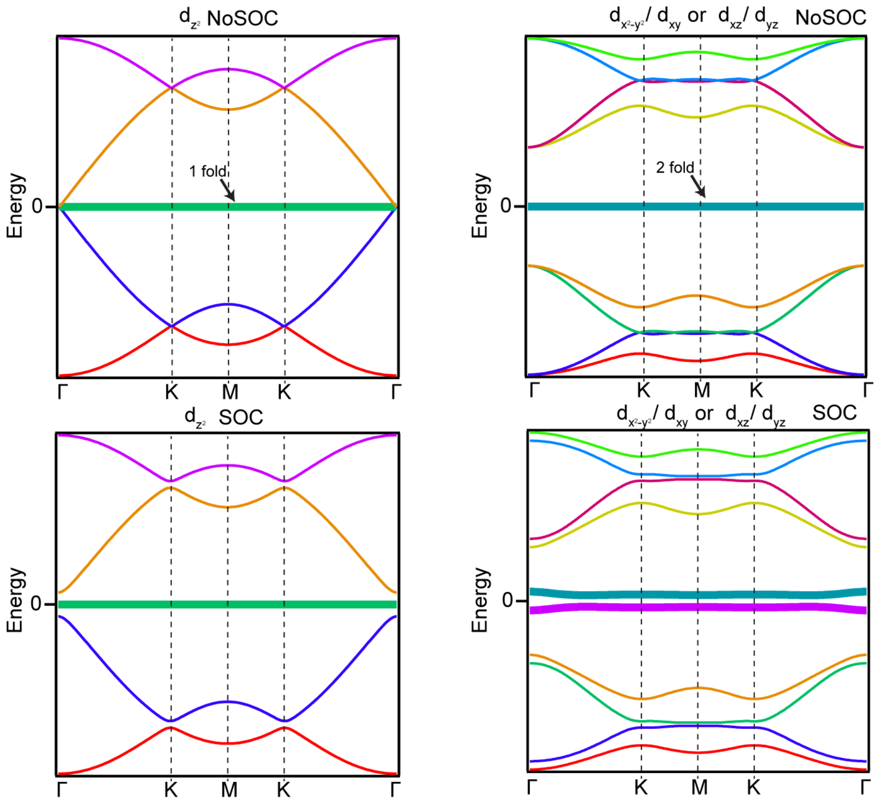

FIG. **Supplementary Figure 9**. **Tight binding model for d orbital clover unit.** Tight binding model for d_{z^2} and d_{xz}/d_{yz} or $d_{x^2-y^2}/d_{xy}$ in a clover unit with and without considering SOC. When the SOC is included, the flat band(s) near E_F are topologically non-trivial. Different color represents different band.

for the single layer $Fe_{5-\delta}GeTe_2$ is important as a starting point.

Symmetry analysis for the single-layer case

The single layer Fe_5GeTe_2 belongs to the space group $P\bar{3}1m$ (SG164), in which Fe and Te atoms are central symmetrically distributed with respect to Ge. Four Fe (Fe(2) and Fe(3)) atoms occupy two pairs of Wyckoff position 2d, while the other Fe (Fe(1)) randomly occupies one site of the Wyckoff position 2c. We take the Ge as the origin of the coordinate. As shown in Supplementary Figure 11, the point group of the single layer system is generated by C_{3z},

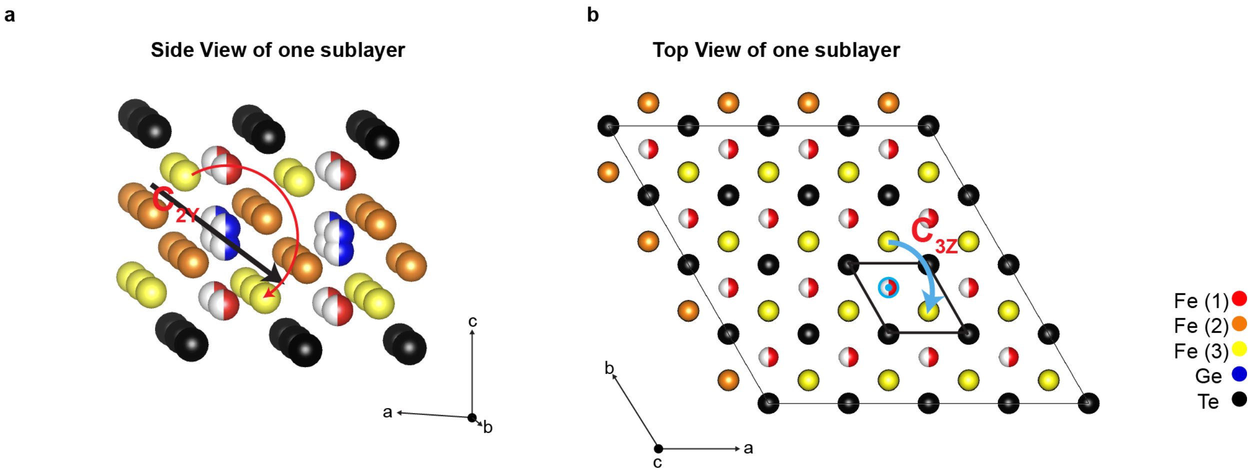

FIG. **Supplementary Figure 10. In slow-cooled samples, the C_{2y} and C_{3z} symmetry. a** Side view of one sublayer, a C_{2y} operation can bring each atom to allowed site. **b** Top view of one sublayer, a C_{3z} operation can bring each atom to allowed site.

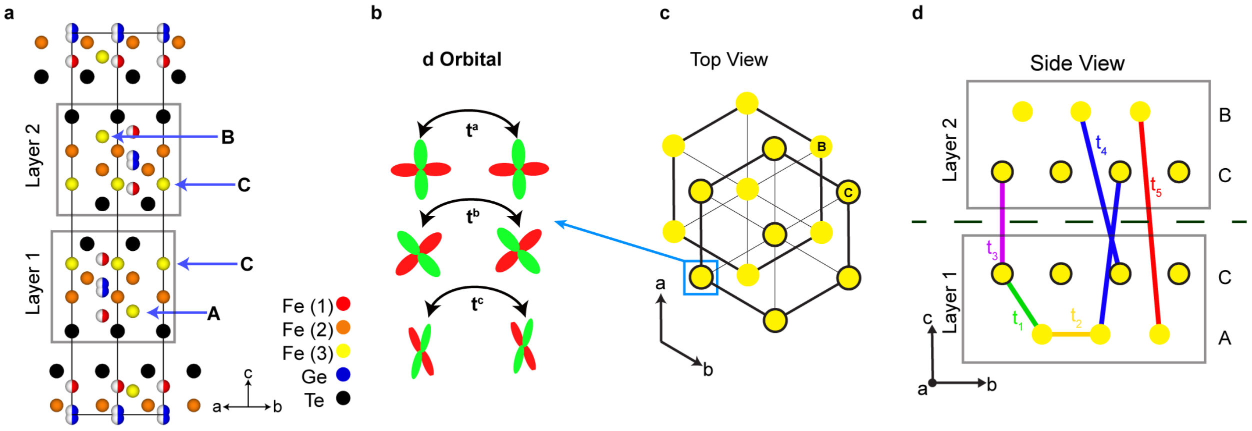

FIG. **Supplementary Figure 11. Tight binding model for d orbital magnetic crossings. a** The described sub-site used to construct the Hamiltonian in the Method. **b** Schematics of the hopping between d orbitals. **c** Top view of the A and B sublayers. **d** Side view of the sublayers and the hopping parameters.

C_{2y} and P, which transforms the coordinate as

$$C_{3z} : (x, y, z) \to (x', y', z), \tag{5}$$

$$C_{2y} : (x, y, z) \to (-x, y, -z), \tag{6}$$

$$P : (x, y, z) \to (-x, -y, -z), \tag{7}$$

with $x' + iy' = e^{i\frac{2\pi}{3}}(x + iy)$. The K point is invariant under C_{3z}, C_{2y} and PT, which allows the existence of 2d irreducible representations.

We first discuss the bands from orbitals of Fe(1) sites. The Fe(1) can be regarded as a stacking of two layers of triangular lattices. The dispersion consists of two copies of bands from the triangular lattice. For a triangular lattice, the band from the d_{z^2} orbital is singly degenerate with an 1d irreducible representation across the whole Brillouin zone. For the $d_{xy}/d_{x^2-y^2}$ and d_{xz}/d_{yz} orbitals, at the K point, they form a 2d irreducible representation and would yield a Dirac crossing. We can distinguish the two cases experimentally by analyzing the ARPES matrix element effect. The band crossing at K is only observed under LV polarized light in the ARPES measurement while not observed under LH polarization. As can be seen in the ARPES setup (Supplementary Figure 12), the LV polarization is completely in-plane and hence cannot probe the out-of-plane d_{z^2} orbital. Therefore we can discard the possibility that the crossing is of d_{z^2} orbital.

We label the rest four Fe atoms from top to bottom as $\mathrm{Fe}^{a=(1-4)}$. $\mathrm{Fe}^1/\mathrm{Fe}^4$ ($\mathrm{Fe}^2/\mathrm{Fe}^3$) constitutes a pair of sites in Wyckoff 2d, dubbed as Fe(2)(Fe(3)). We construct the bases according to the angular momentum for each site as:

$$
\begin{aligned}
|L = \pm 0\rangle_a &= |d_{z^2}\rangle, (a = 1/4, 2/3), \\
|L = \pm 1\rangle_a &= \frac{1}{\sqrt{2}} \left(|d_{xz}\rangle \pm i|d_{yz}\rangle\right), (a = 1/4, 2/3), \\
|L = \pm 2\rangle_a &= \frac{1}{\sqrt{2}} \left(|d_{x^2-y^2}\rangle \pm i|d_{xy}\rangle\right), (a = 1/4, 2/3),
\end{aligned}
\tag{8}
$$

where L denotes the orbital angular momentum. Because Fe(2) and Fe(3) are two sets of Wyckoff position 2d, we pick Fe(3) as the example for further symmetry analysis. In a real system, the band structure is a linear mixing of these two set of orbitals. The transformation of these bases at K point are summarized in Table I, in which the phase $\phi = \boldsymbol{k} \cdot \boldsymbol{a}$ is related to the relative position of the sublattice.

	C_{3z}	C_{2y}/PT
$\lvert \pm L\rangle_2$	$e^{\pm i\frac{2L\pi}{3}} e^{i\phi}\lvert \pm L\rangle_2$	$\lvert \mp L\rangle_3$
$\lvert \pm L\rangle_3$	$e^{\pm i\frac{2L\pi}{3}} e^{-i\phi}\lvert \pm L\rangle_3$	$\lvert \mp L\rangle_2$

Supplementary Table I. The additional phase $\phi = \frac{4\pi}{3}$ is because of the lattice position, $e^{i\boldsymbol{k}\boldsymbol{a}} = e^{i\frac{4\pi}{3}}$. The phase $\phi = \boldsymbol{k} \cdot \boldsymbol{a}$ is related to the relative position of the sublattice

For $L = 0$, the C_{3z} rotation transforms the sublattice back to itself while C_{2y} symmetry connects the orbitals from different sublattices. This is reminiscent to the s-orbital honeycomb

model, in which the bands cross at the K point and form a 2d irreducible representation. For the $L \neq 0$ case, the bands at the K point are consist of two 1d irreducible representations and one 2d irreducible representation as $A_1 + A_2 + E$.

We further consider the effect of the spin-orbit coupling. We consider the on-site Dresselhaus-type spin-orbit coupling described by Hamiltonian $H_{SO} = \lambda_{SO}\boldsymbol{L} \cdot \boldsymbol{S}$, where λ_{SO} is the strength of SOC, $\boldsymbol{L}$ and $\boldsymbol{S}$ are the orbital and spin angular momenta, respectively. The ferromagnetism with $\langle \boldsymbol{S} \neq 0 \rangle$ effectively contributes a Zeeman term in the orbital basis as $H_{SO} \approx \lambda_{SO}\boldsymbol{L} \cdot \langle \boldsymbol{S} \rangle$. For the $L = 0$ case, $H_{SO} = 0$, the crossing at the K point is stable against SOC. While for $L \neq 0$, the crossing at the K point is gapped by the SOC and releases a large Berry curvature distribution. We do note that anomalous Hall effect has been observed in $Fe_{5-\delta}GeTe_2$ [1], which may be consistent with the contribution of Berry curvature from the gapped ferromagnetic Dirac nodal line, though the firm establishment of this connection still awaits future work.

Effective model for the single-layer case

We next construct the tight binding model to describe the band structure for $L \neq 0$. Since the construction of the $L = 1$ and $L = 2$ are similar, we take $d_{xy}/d_{x^2-y^2}$ as example for further illustration. This model follows all the symmetry constraints of the single layer $Fe_{5-\delta}GeTe_2$ with orbitals from one set of Wyckoff position 2d. The Hamiltonian takes the form as $\mathcal{H} = \Psi^\dagger(k)H_{2D}(k)\Psi(k)$, with $\Psi(k) = [c_{A_1}(k), c_{A_2}(k), c_{B_1}(k), c_{B_2}(k)]^T$, where A/B and $1/2$ denote the sublattices and $d_{x^2-y^2}/d_{xy}$ orbitals, respectively.

$$H_{2D}(k) = \begin{pmatrix} T_2^{11} & T_2^{12} & T_1^{11} & T_1^{12} \\ T_2^{21} & T_2^{22} & T_1^{21} & T_1^{22} \\ T_1^{11*} & T_1^{21*} & T_2^{11} & T_2^{21} \\ T_1^{12*} & T_1^{22*} & T_2^{12} & T_2^{22} \end{pmatrix}, \quad (9)$$

with the elements of the nearest neighbor hoppings:

$$\begin{aligned} T_1^{11} &= t_1^a e^{i\boldsymbol{k}\boldsymbol{\delta}_1} + \frac{3t_1^b + t_1^a}{4}(e^{i\boldsymbol{k}\boldsymbol{\delta}_2} + e^{i\boldsymbol{k}\boldsymbol{\delta}_3}) \\ T_1^{12} = T_1^{21} &= -\frac{\sqrt{3}}{4}(t_1^a - t_1^b)(e^{i\boldsymbol{k}\boldsymbol{\delta}_2} - e^{i\boldsymbol{k}\boldsymbol{\delta}_3}) \\ T_1^{22} &= t_1^b e^{i\boldsymbol{k}\boldsymbol{\delta}_1} + \frac{t_1^b + 3t_1^a}{4}(e^{i\boldsymbol{k}\boldsymbol{\delta}} + e^{i\boldsymbol{k}\boldsymbol{\delta}_3}), \end{aligned} \tag{10}$$

and the elements for the next nearest neighbor hoppings:

$$\begin{aligned} T_2^{11} &= t_2^a \cos \boldsymbol{k}(\boldsymbol{\delta}_2 - \boldsymbol{\delta}_3) + \frac{3t_2^b + t_2^a}{4}\left(\cos \boldsymbol{k}(\boldsymbol{\delta}_2 - \boldsymbol{\delta}_1) + \cos \boldsymbol{k}(\boldsymbol{\delta}_3 - \boldsymbol{\delta}_1)\right) \\ T_2^{12} = T_2^{21} &= -\frac{\sqrt{3}}{4}(t_2^a - t_2^b)(\cos \boldsymbol{k}(\boldsymbol{\delta}_2 - \boldsymbol{\delta}_1) - \cos \boldsymbol{k}(\boldsymbol{\delta}_3 - \boldsymbol{\delta}_1)) \\ T_2^{22} &= t_2^b \cos \boldsymbol{k}(\boldsymbol{\delta}_2 - \boldsymbol{\delta}_3) + \frac{t_2^b + 3t_2^a}{4}\left(\cos \boldsymbol{k}(\boldsymbol{\delta}_2 - \boldsymbol{\delta}_1) + \cos \boldsymbol{k}(\boldsymbol{\delta}_3 - \boldsymbol{\delta}_1)\right), \end{aligned} \tag{11}$$

where $\boldsymbol{\delta}_1 = (-\frac{1}{\sqrt{3}}, 0)$, $\boldsymbol{\delta}_2/\boldsymbol{\delta}_3 = (\frac{1}{2\sqrt{3}}, \pm\frac{1}{2})$ are vectors connects nearest neighbor A and B sublattices. The t^a and t^b is the direct hopping between $d_{x^2-y^2} - d_{x^2-y^2}$ and $d_{xy} - d_{xy}$ orbitals, as described in Supplementary Figure 11b.

We now include the spin-orbit coupling. When the ferromagnetism is along z direction, $H_{SO} \propto \tau_z$ in the $|L = \pm 2\rangle$ bases or $H_{SO} = \Delta\tau_y$ in the effective d_{xy}, $d_{x^2-y^2}$ bases. We now include it in the effective tight binding model as we proposed in Eq. 9, the additional term described the spin-orbit coupling is:

$$H_{SO}(k) = \Delta_{SO} \begin{pmatrix} 0 & 2j & 0 & 0 \\ -2j & 0 & 0 & 0 \\ 0 & 0 & 0 & 2j \\ 0 & 0 & -2j & 0 \end{pmatrix}. \tag{12}$$

The factor 2 is coming from the angular momentum $L = \pm 2$. The SOC generically gaps out the Dirac crossing and contributes nontrivial Berry curvature.

Symmetry and the effective model of the ABC-stacked case

We next consider the symmetry constraints in the 3D bulk system. Before the analysis of the ABC stacking of layers, we first examine the symmetry of a simpler AAA stacking. The

hopping along z direction extends the 2D hexagonal Brillouin zone (BZ) into 3D hexagonal prism. At arbitrary momentum along the KH line away from K and H point, C_{2y} is no longer a valid symmetry, while the crossing can still be preserved by C_{3z} and PT symmetries. We next turn to the ABC stacking of the layers. The system now belongs to a rhombohedral space group $R\bar{3}m$ (No. 166), in whose BZ, the KH is no longer a high symmetry line. As been proposed in other context of the ABC stacked materials like the rhombohedral graphite or Fe_3Sn_2, without spin-orbit coupling, a helical nodal line can be realized if the interlayer hopping is relatively smaller compared with the intralayer hoppings [4].

In order to capture this helical nodal line in the bulk of $Fe_{5-\delta}GeTe_2$, we stack the previously proposed 2D d-orbital honeycomb model in a rhombohedral fashion and construct a 3D tight binding. The Hamiltonian is

$$H_{3D}(k) = \begin{pmatrix} H_{2D}(k) & H_T(k) & H_T^\dagger(k) \\ H_T^\dagger(k) & H_{2D}(k) & H_T(k) \\ H_T(k) & H_T^\dagger(k) & H_{2D}(k) \end{pmatrix}, \tag{13}$$

where $H_{2D}(k)$ follows the $2D$ Hamiltonian defined in Eq. 9. The connecting Hamiltonian

$$H_T(k) = \begin{pmatrix} T'^{11}_{mm} & T'^{12}_{mm} & T'^{11}_{mn} & T'^{12}_{mn} \\ T'^{21}_{mm} & T'^{22}_{mm} & T'^{21}_{mn} & T'^{22}_{mn} \\ T'^{11*}_{mn} & T'^{21*}_{mn} & T'^{11}_{nn} & T'^{21}_{nn} \\ T'^{12*}_{mn} & T'^{22*}_{mn} & T'^{12}_{nn} & T'^{22}_{nn} \end{pmatrix}, \tag{14}$$

where m/n denotes the two different sublattice in the rhombohedral unit cell, and

$$
\begin{aligned}
T'^{11}_{mm} = T'^{11}_{nn} &= t_4^a \cos(\boldsymbol{k}_\perp \cdot \boldsymbol{\delta}_1 + k_z c) + \frac{3t_4^b + t_4^a}{4} \left[\cos(\boldsymbol{k}_\perp \cdot \boldsymbol{\delta}_2 + k_z c) + \cos(\boldsymbol{k}_\perp \cdot \boldsymbol{\delta}_1 + k_z c)\right] \\
T'^{22}_{mm} = T'^{22}_{nn} &= t_4^b \cos(\boldsymbol{k}_\perp \cdot \boldsymbol{\delta}_1 + k_z c) + \frac{3t_4^a + t_4^b}{4} \left[\cos(\boldsymbol{k}_\perp \cdot \boldsymbol{\delta}_2 + k_z c) + \cos(\boldsymbol{k}_\perp \cdot \boldsymbol{\delta}_1 + k_z c)\right] \\
T'^{12}_{mm} = T'^{21}_{mm} = = T'^{12}_{nn} = T'^{21}_{nn} &= -\frac{\sqrt{3}}{4}(t_4^a - t_4^b)(\cos[\boldsymbol{k}_\perp(\boldsymbol{\delta}_2 - \boldsymbol{\delta}_1) + k_z c] - \cos[\boldsymbol{k}_\perp(\boldsymbol{\delta}_3 - \boldsymbol{\delta}_1) + k_z c]) \\
T'^{11}_{mn} &= t_3^c e^{ik_z c} + t_5^a e^{i(-\boldsymbol{k}_\perp \cdot \boldsymbol{\delta}_1 + k_z c)} + \frac{3t_5^b + t_5^a}{4}(e^{i(-\boldsymbol{k}_\perp \cdot \boldsymbol{\delta}_2 + k_z c)} + e^{i(-\boldsymbol{k}_\perp \cdot \boldsymbol{\delta}_3 + k_z c)}) \\
T^{12}_{mn} = T^{21}_{mn} = T^{21}_{1} &= -\frac{\sqrt{3}}{4}(t_5^a - t_5^b)(e^{i(-\boldsymbol{k}_\perp \cdot \boldsymbol{\delta}_2 + k_z c)} - e^{(-i\boldsymbol{k} \cdot \boldsymbol{\delta}_3 + k_z c)}) \\
T'^{22}_{mn} &= t_3^c e^{ik_z c} + t_5^b e^{i(-\boldsymbol{k}_\perp \cdot \boldsymbol{\delta}_1 + k_z c)} + \frac{3t_5^a + t_5^b}{4}(e^{i(-\boldsymbol{k}_\perp \cdot \boldsymbol{\delta}_2 + k_z c)} + e^{i(-\boldsymbol{k}_\perp \cdot \boldsymbol{\delta}_3 + k_z c)}),
\end{aligned}
\tag{15}
$$

where $\boldsymbol{k}_\perp = (k_x, k_y)$. $H_T^\dagger(k)$ is the Hermitian conjugate of the matrix $H_T(k)$. Besides t^a and t^b, we further include t^c to denote the face-to-face hopping between $d_{x^2-y^2}$ (d_{xy}). The distances $1-5$ are explicitly described in Supplementary Figure 11. As the strength of the warping away from the K point depends on the layer-layer interaction, the weak interlayer coupling in the vdW compounds prevent us from resolving the small warping of the nodal lines.

SUPPLEMENTARY NOTE 10: DESTRUCTIVE INTERFERENCE AND EFFECTIVE MODEL FOR THE SITE-ORDERED SAMPLE

In this section, we consider the case when the positions of the Fe(1) atoms are fixed and develop a $\sqrt{3} \times \sqrt{3}$ lattice order.

Flat band for the $L = 0$ orbital

We first discuss the bands from d_{z^2} orbitals and only consider the nearest neighbor hoping between the red and yellow sites t_1 as shown in Fig. 4h. The Hamiltonian takes the simple form as:

$$
H(k_x, k_y) = \begin{pmatrix} 0 * \mathbb{1}_{2\times 2} & \mathcal{H}(k_x, k_y) \\ \mathcal{H}^\dagger(k_x, k_y) & 0 * \mathbb{1}_{3\times 3} \end{pmatrix} \tag{16}
$$

where $\mathcal{H}^\dagger$ is the Hermitian conjugate of the matrix $\mathcal{H}$, and

$$\mathcal{H}(k_x, k_y) = t_1 \begin{pmatrix} e^{i(\frac{\sqrt{3}}{6}k_x+\frac{1}{6}k_y)} & e^{-i(\frac{\sqrt{3}}{6}k_x+\frac{1}{6}k_y)} & e^{-i\frac{1}{3}k_y} \\ e^{i(-\frac{\sqrt{3}}{6}k_x+\frac{1}{6}k_y)} & e^{-i\frac{1}{3}k_y} & e^{i(\frac{\sqrt{3}}{6}k_x+\frac{1}{6}k_y)} \end{pmatrix}. \tag{17}$$

One can easily diagonalize the Hamiltonian and the dispersion is shown in Supplementary Figure 9. The flat band is single degenerate and the corresponding localized wave function for the flat band is:

$$\begin{pmatrix} 0 & 0 & e^{\frac{k_y}{3}} - e^{-\frac{2}{3}k_y} & e^{-\frac{k_x}{2\sqrt{3}}-\frac{k_y}{6}} - e^{\frac{k_x}{\sqrt{3}}+\frac{k_y}{3}} & e^{\frac{k_x}{2\sqrt{3}}-\frac{k_y}{6}} - e^{-\frac{k_x}{\sqrt{3}}+\frac{k_y}{3}} \end{pmatrix}. \tag{18}$$

The real-space orbital textures for the flat band eigenstate is shown in Fig. 4.

The on-site SOC described by $\boldsymbol{L} \cdot \boldsymbol{S}$ will not influence the obtained band structure because $\boldsymbol{L} = 0$ for d_{z^2} orbitals. While one can still introduce long-range SOC without further breaking any symmetries. We consider the nearest spin-orbit coupling which takes the form as:

$$H_{soc}(k_x, k_y) = \begin{pmatrix} 0 * \mathbb{1}_{2\times 2} & 0 * \mathbb{1}_{2\times 3} \\ 0 * \mathbb{1}_{3\times 2} & \mathcal{H}_{soc}(k_x, k_y) \end{pmatrix}, \tag{19}$$

with

$$\mathcal{H}_{soc}(k_x, k_y) = \lambda_{soc} \begin{pmatrix} 0 & e^{-i(\frac{\sqrt{3}}{6}k_x+\frac{1}{2}k_y)} & e^{i(\frac{\sqrt{3}}{6}k_x+\frac{1}{2}k_y)} \\ e^{i(\frac{\sqrt{3}}{6}k_x+\frac{1}{2}k_y)} & 0 & e^{i\frac{3}{3}k_x} \\ e^{-i(\frac{\sqrt{3}}{6}k_x-\frac{1}{2}k_y)} & e^{-i\frac{3}{3}k_x} & 0 \end{pmatrix} \tag{20}$$

with λ_{soc} the strength of the spin-orbit coupling. As shown in Supplementary Figure 9, the spin orbit coupling gaps out the node at Γ and K. The isolated band is topologically nontrivial.

Flat band for the $L \neq 0$ orbital

We next consider the case with $L \neq 0$. For illustration, we take d_{xz}/d_{yz} with $|L| = 1$ as example. The Hamiltonian can be written in the basis

$$[c_{A_1}(\boldsymbol{k}), c_{B_1}(\boldsymbol{k}), c_{C_1}(\boldsymbol{k}), c_{D_1}(\boldsymbol{k}), c_{E_1}(\boldsymbol{k}), c_{A_2}(\boldsymbol{k}), c_{B_2}(\boldsymbol{k}), c_{C_2}(\boldsymbol{k}), c_{D_2}(\boldsymbol{k}), c_{E_2}(\boldsymbol{k})] \tag{21}$$

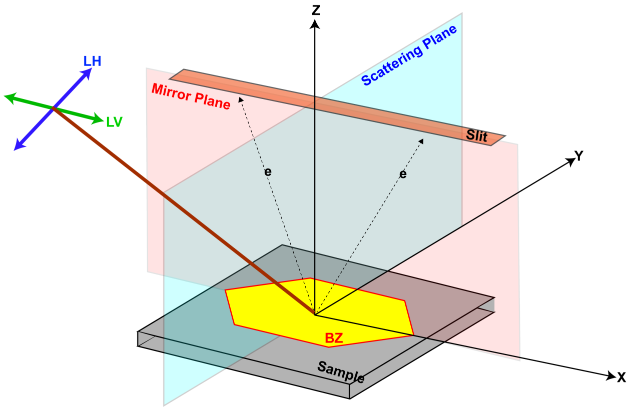

FIG. **Supplementary Figure 12**. **ARPES experimental setup.** The measured geometry is shown here with the BZ outlined by red lines. The analyzer slit is aligned to the $\Gamma - K$ direction of the sample. LV polarization is completely in-plane while LH polarization contains a finite out-of-plane component.

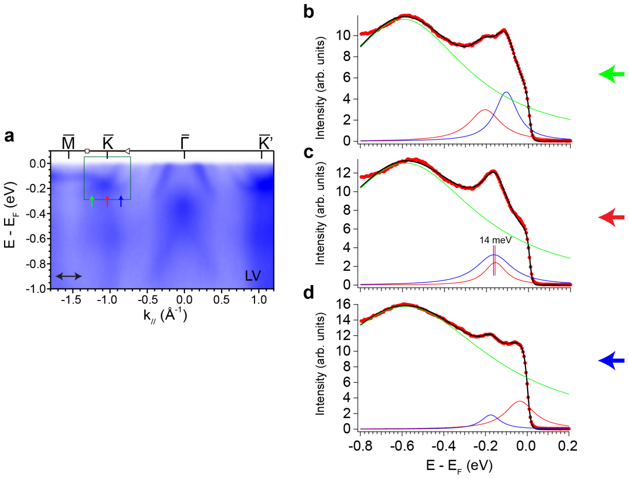

FIG. **Supplementary Figure 13**. **Topological nodal line gap fitting: Estimation of SOC gap for site-disordered phase. a** High symmetry cut spectra measured in site-disordered phase. The crossing at the K point is marked by red arrow. **b-d** EDCs taken at the three colored arrows shown in **a** together with the fitting of the two Lorentzian peaks (red and blue) on top of a Gaussian background (green) multiplied by a Fermi-Dirac distribution function. The energy resolution was 26 meV.

where 1/2 and A-E denote the d_{xz}/d_{yz} and sublattices, respectively. And

$$H(k_x, k_y) = \begin{pmatrix} 0 * \mathbb{1}_{2\times 2} & \mathcal{H}_{11} & 0 * \mathbb{1}_{2\times 2} & \mathcal{H}_{12} \\ \mathcal{H}_{11}^* & 0 * \mathbb{1}_{3\times 3} & \mathcal{H}_{21}^* & 0 * \mathbb{1}_{3\times 3} \\ 0 * \mathbb{1}_{2\times 2} & \mathcal{H}_{21} & 0 * \mathbb{1}_{2\times 2} & \mathcal{H}_{22} \\ \mathcal{H}_{12}^* & 0 * \mathbb{1}_{3\times 3} & \mathcal{H}_{22}^* & 0 * \mathbb{1}_{3\times 3} \end{pmatrix} \tag{22}$$

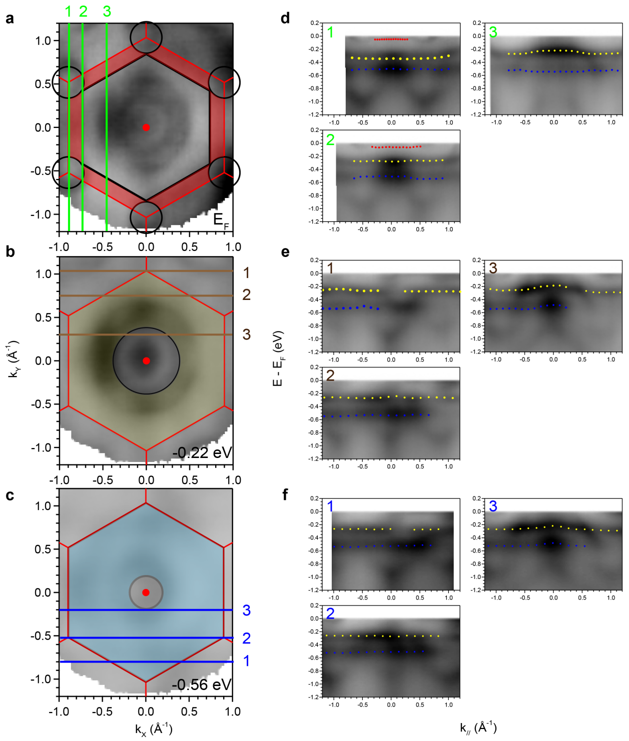

FIG. **Supplementary Figure 14**. **Flatness estimation for site-ordered phase. a-c** Constant energy contours at the energy of the flat dispersions. Shaded regions indicate the portion of the BZ where each flat band is within 50 meV window. **d-f** Dispersions measured along indicated cuts, where the dots are the fitted positions of the band dispersions. Red is associated with the flat band near EF. Yellow is associated with the flat band near -0.22eV. Blue is associated with the flat band near -0.56 eV.

with

$$\mathcal{H}_{ab} = \begin{pmatrix} t_{ab}^{AC} e^{i\boldsymbol{k}(\delta_A-\delta_C)} & t_{ab}^{AD} e^{i\boldsymbol{k}(\delta_A-\delta_D)} & t_{ab}^{AE} e^{i\boldsymbol{k}(\delta_A-\delta_E)} \\ t_{ab}^{BC} e^{i\boldsymbol{k}(\delta_B-\delta_C)} & t_{ab}^{BD} e^{i\boldsymbol{k}(\delta_B-\delta_D)} & t_{ab}^{BE} e^{i\boldsymbol{k}(\delta_B-\delta_E)} \end{pmatrix}, \tag{23}$$

where δ_αs ($\alpha = A \sim E$) are the positions of the sublattices. And the coupling strength $t_{ab}^{\alpha\beta}$s follow the Table. II, in which t_x and t_y represent the direct hoppings between $d_{xz} - d_{xz}$

ab \ $\alpha\beta$	AC	AD	AE	BC	BD	BE
11	$\frac{3}{4}t_x + \frac{1}{4}t_y$	$\frac{3}{4}t_x + \frac{1}{4}t_y$	t_y	$\frac{3}{4}t_x + \frac{1}{4}t_y$	t_y	$\frac{3}{4}t_x + \frac{1}{4}t_y$
12	$\frac{\sqrt{3}}{4}t_x - \frac{\sqrt{3}}{4}t_y$	$-\frac{\sqrt{3}}{4}t_x + \frac{\sqrt{3}}{4}t_y$	0	$-\frac{\sqrt{3}}{4}t_x + \frac{\sqrt{3}}{4}t_y$	0	$\frac{\sqrt{3}}{4}t_x - \frac{\sqrt{3}}{4}t_y$
21	$\frac{\sqrt{3}}{4}t_x - \frac{\sqrt{3}}{4}t_y$	$-\frac{\sqrt{3}}{4}t_x + \frac{\sqrt{3}}{4}t_y$	0	$-\frac{\sqrt{3}}{4}t_x + \frac{\sqrt{3}}{4}t_y$	0	$\frac{\sqrt{3}}{4}t_x - \frac{\sqrt{3}}{4}t_y$
22	$\frac{1}{4}t_x + \frac{3}{4}t_y$	$\frac{1}{4}t_x + \frac{3}{4}t_y$	t_x	$\frac{1}{4}t_x + \frac{3}{4}t_y$	t_x	$\frac{1}{4}t_x + \frac{3}{4}t_y$

Supplementary Table II. The hopping table of $t_{ab}^{\alpha\beta}$, where $a/b = 1/2$ denotes the orbitals and $\alpha\beta = A \sim E$ denotes the sublattices.

and $d_{yz} - d_{yz}$. The geometric destructive flat band is shown in Supplementary Figure 9. A

distinct difference from the d_{z^2} orbitals is that the flat band is doubly degenerate.

We further consider the influence from the spin orbit coupling. The $\boldsymbol{L} \cdot \boldsymbol{S}$ type of the SOC takes the form as

$$H_{soc} = \lambda_{soc} \tau_y \otimes \mathbb{1}_{5\times 5}, \tag{24}$$

with τ_y the Pauli matrices in the orbital basis. As shown in Supplementary Figure 9, the degenerate flat band splits into two with spin orbit coupling. Both bands are topologically non-trivial.

I. SUPPLEMENTARY REFERENCES

[1] May, A. F., Bridges, C. A. & McGuire, M. A. Physical properties and thermal stability of $Fe_{5-x}GeTe_2$ single crystals. *Phys. Rev. Mater.* **3**, 104401 (2019).

[2] May, A. F. *et al.* Ferromagnetism near room temperature in the cleavable van der Waals crystal Fe_5GeTe_2. *ACS Nano* **13**, 4436–4442 (2019).

[3] Ly, T. T. *et al.* Direct observation of Fe−Ge ordering in $Fe_{5-x}GeTe_2$ crystals and resultant helimagnetism. *Adv. Funct. Mater.* **31**, 2009758 (2021).

[4] Fang, S. *et al.* Ferromagnetic helical nodal line and kane-mele spin-orbit coupling in kagome metal Fe_3Sn_2. *Phys. Rev. B* **105**, 035107 (2022).
